# Mobile Phone Apps to Promote Weight Loss and Increase Physical Activity: A Systematic Review and Meta-Analysis

**DOI:** 10.2196/jmir.4836

**Published:** 2015-11-10

**Authors:** Gemma Flores Mateo, Esther Granado-Font, Carme Ferré-Grau, Xavier Montaña-Carreras

**Affiliations:** ^1^ Institut Universitari d'Investigació en Atenció Primària (IDIAP) Jordi Gol Reus Spain; ^2^ Institut Català de la Salut Centre Atenció Primària Horts de Miró Reus Spain; ^3^ Universitat Rovira i Virgili Tarragona Spain

**Keywords:** mHealth, mobile phone, apps, obesity, physical activity, intervention

## Abstract

**Background:**

To our knowledge, no meta-analysis to date has assessed the efficacy of mobile phone apps to promote weight loss and increase physical activity.

**Objective:**

To perform a systematic review and meta-analysis of studies to compare the efficacy of mobile phone apps compared with other approaches to promote weight loss and increase physical activity.

**Methods:**

We conducted a systematic review and meta-analysis of relevant studies identified by a search of PubMed, the Cumulative Index to Nursing and Allied Health Literature (CINAHL), and Scopus from their inception through to August 2015. Two members of the study team (EG-F, GF-M) independently screened studies for inclusion criteria and extracted data. We included all controlled studies that assessed a mobile phone app intervention with weight-related health measures (ie, body weight, body mass index, or waist circumference) or physical activity outcomes. Net change estimates comparing the intervention group with the control group were pooled across studies using random-effects models.

**Results:**

We included 12 articles in this systematic review and meta-analysis. Compared with the control group, use of a mobile phone app was associated with significant changes in body weight (kg) and body mass index (kg/m^2^) of -1.04 kg (95% CI -1.75 to -0.34; I2 = 41%) and -0.43 kg/m^2^ (95% CI -0.74 to -0.13; I2 = 50%), respectively. Moreover, a nonsignificant difference in physical activity was observed between the two groups (standardized mean difference 0.40, 95% CI -0.07 to 0.87; I2 = 93%). These findings were remarkably robust in the sensitivity analysis. No publication bias was shown.

**Conclusions:**

Evidence from this study shows that mobile phone app-based interventions may be useful tools for weight loss.

## Introduction

Overweight and obesity are a global public health issue and an important feature in discussions about strategies for primary and secondary health care. Developing since the 1960s and now gathering pace rapidly, the issue is contributing, together with population aging­­, to major increases in the prevalence of high blood pressure and cholesterol levels, type 2 diabetes, and cancers [1]. Mortality rates are increasing with increasing degrees of overweight, as measured by body mass index (BMI) [2].

In 2008, 35% of adults older than 20 years were overweight (BMI ≥ 25 kg/m^2^) and the worldwide prevalence of obesity (BMI ≥30 kg/m^2^) had nearly doubled since 1980, from 5% of men and 8% of women to 10% and 14%, respectively [2]. An estimated 205 million men and 297 million women were obese—a total of more than half a billion adults worldwide [3]. For these reasons, identifying effective interventions is an important component in public health efforts to curb obesity, but the most effective strategies for weight loss remain unclear.

With the extensive market penetration of mobile phones, the International Telecommunications Union (ITU) reports that as of 2015, there are more than 7 billion mobile cellular subscriptions worldwide, corresponding to a 97% penetration rate—defined by ITU as mobile cellular telephone subscribers per 100 inhabitants [4]. Advanced-feature mobile phones (those with computer operating systems) have broadened the functions of mobile phones considerably. Mobile phone apps meet a variety of user needs, and are designed and adapted for each type of mobile device; therefore, they are applicable in nearly all social and economic sectors and environments. At present, these apps, apart from their recreational function, are becoming instruments of patient education and support and are also helpful to health care professionals [5]. Nonetheless, the market for health care apps is very fragmented because many of them are very specific or directed at minority diseases or specialties. The world market for medical apps for mobile phones and tablets multiplied seven times over in 2011 alone, reaching a total of US $718 million according to a market analysis by the American firm research2guidance [6]. A recent analysis of app store catalogs identified more than 97,000 mHealth apps, most of them dealing with general health and physical fitness; in general, they facilitate the monitoring of various parameters by individual users and provide general information and support related to those topics [5]. Previous research has suggested that mobile apps may be beneficial in asthma control [7] and diabetes management [8,9].

To our knowledge, no meta-analysis to date has assessed the efficacy of mobile phone apps to promote weight loss and increase physical activity. The objective of this study was to perform a systematic review and meta-analysis of published studies to evaluate the efficacy of interventions that included mobile phone apps compared with other interventions to reduce weight and increase physical activity in populations of children and adults.

## Methods

### Search Strategy

We conducted a systematic literature search of three databases from their inception through August 30, 2015, to identify studies examining the effectiveness of a mobile app intervention compared with a control intervention in achieving anthropometric or physical activity changes: Medline (via PubMed; National Library of Medicine, Bethesda, MD; started in 1966), Scopus (Elsevier; started in 1995), and the Cumulative Index to Nursing and Allied Health Literature (CINAHL; started in 1960). Details on the search strategy are presented in Multimedia Appendix 1. Briefly, our literature search strategy combined synonyms for mobile app (the intervention of interest) with synonyms for the three outcomes: weight, body mass index, and exercise. The search period was all-inclusive up to August 2015. There were no language restrictions. In addition, we manually reviewed reference lists from relevant original research and review articles.

### Study Selection

**Figure 1:**
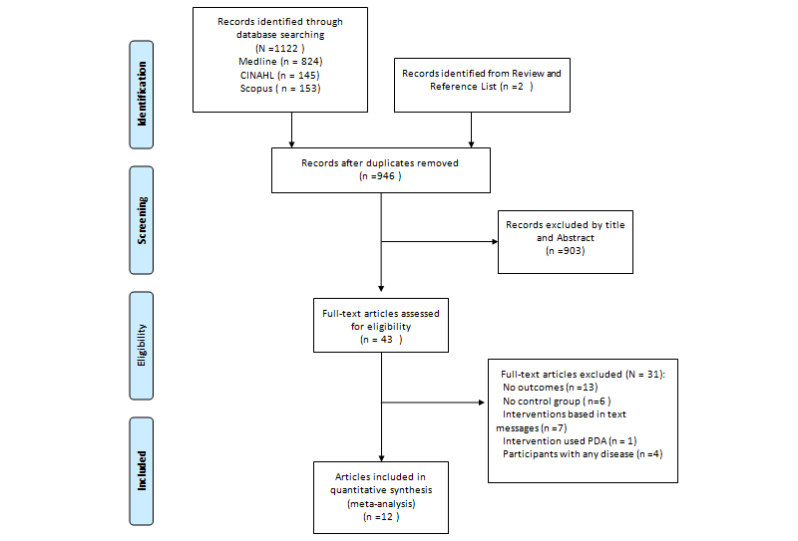
Flowchart for the selection of the articles in this meta-analysis.

Two members of the study team (EG-F, GF-M) independently screened studies for inclusion criteria and extracted the data. We included all studies that assessed a mobile app intervention, compared to a control group, with weight-related health measures (ie, body weight, BMI, or waist circumference) or physical activity outcomes. We included studies performed in populations of children and of adults. Exclusion criteria were as follows: (1) no original research (ie, reviews, editorials, or nonresearch letters), (2) case reports and case series, (3) data on body weight, BMI, waist circumference, or physical activity not reported, (4) no control group, (5) participants with any disease except a diagnosis of obesity, (6) mobile telephone intervention based on text messaging, such as short message service (SMS), and (7) intervention used or included personal digital assistants (PDAs). Ethics approval was not required because only published data were analyzed in this review. The study selection process is summarized in Figure 1.

### Data Extraction and Quality Assessment

Two investigators (EG-F, GF-M) independently abstracted articles that met the selection criteria and resolved discrepancies by consensus. A form developed in Microsoft Word was used to extract data from eligible research papers, including author, country of study, age of participants, length of follow-up, sample size, and study outcomes. Study outcomes recorded were mean and standard deviation (SD) body weight, BMI, waist circumference, and/or physical activity. These values were captured as mean changes from baseline to the end of the intervention, with variations reported as SD, standard error (SE), or 95% CI. When there were several publications from the same cohort, the study with the longest follow-up was selected; when the follow-up was equivalent, we selected the study with the largest number of cases, the publication that used internal comparisons, or the most recent study. An intention-to-treat analysis was used wherever possible. The risk of bias was assessed following Cochrane recommendations, considering random sequence generation, allocation concealment, blinding of participants and personnel, blinding of outcome assessment, incomplete outcome data, and selective reporting [10]. Each criterion was categorized as *clearly yes*, *not sure*, or *clearly no*. Criteria for which there were differences between the two evaluators were discussed until a consensus decision was reached.

### Statistical Methods

For each study, the net effect size was calculated as the change in body weight-related and physical activity measures resulting from treatment from baseline to the end of the intervention in the intervention group, minus the change in body weight-related and physical activity measures in the control group during the same time period. The SEs and CIs were converted to SDs for analysis. For studies without SD data, we calculated the variance from CIs or test statistics. If the SD for change between baseline and the end of the intervention was not reported, it was calculated using the following equation [11]:

SD^2^_diff_= SD^2^_pre_+ SD^2^_post_- 2×ρ×SD_pre_×SD_post_

Where SD_pre_corresponds to the SD at baseline, SD_post_corresponds to the SD at the end of intervention, and ρ is the correlation coefficient for correlations between measurements taken at baseline and at the end of the intervention.

For body weight and BMI, weighted mean differences (WMDs) were estimated using random-effects models. For physical activity outcomes, standardized mean differences (SMDs) were estimated using random-effects models. Heterogeneity was quantified with the I2 statistic, which describes the proportion of total variation in study estimates as a result of heterogeneity [12]. To further assess the robustness of our findings, we performed several sensitivity analyses by excluding nonrandomized studies, or studies that did not report the intervention in the control group. We also assessed the relative influence of each study on pooled estimates by omitting one study at a time. Finally, we assessed the publication bias by using Egger's test and funnel plots. Statistical analyses were performed with Review Manager software, version 5.3 (The Nordic Cochrane Centre, The Cochrane Collaboration).

## Results

### Study Selection

The search strategy retrieved 1124 articles from different sources and 12 articles were included in this meta-analysis [13-24] (see Figure 1 and Table 1). One study contributed two articles [14,15]. We used BMI data from the 2011 Turner-McGrievy and Tate study [14], but because that report did not include physical activity measurements, we took the physical activity data from a 2013 publication by Turner-McGrievy et al [15]. Studies were published between 2010 and 2015, and sample sizes ranged from 35 [18] to 361 [22]. There were two nonrandomized controlled trials [13,16], but the rest of the studies were randomized controlled trials. The interventions in many control groups were ones such as traditional interventions or intensive counseling. Only one study did not specify the type of intervention in the control group [13] (see Table 2).

**Table 1:** Characteristics of included clinical trials.

Author, year	Country	Study design	Population	SS^a^	Men, %	Age in years, mean	Study duration	Outcome
Lee, 2010 [13]	South Korea	CCS^b^	Voluntary participants from the obese clinic at the fitness center, Seoul	36	N/A^c^	28.5	6 weeks	Body weight, BMI^d^
Turner-McGrievy, 2011 [14], 2013 [15]	United States	RCT^e^	Overweight and obese men and women	96	24.7	44	6 months	BMI, physical activity
Kirwan, 2012 [16]	Australia	MCCT^f^	General population	200	52	40	3 months	Physical activity
Carter, 2013 [17]	United Kingdom	RCT	Overweight volunteers	86	33	41.2	6 months	Body weight, BMI
Allen, 2013 [18]	United States	RCT	Overweight and obese men and women	35	22.1	44.9	6 months	Body weight, BMI, waist circumference
Brindal, 2013 [19]	Australia	RCT	Adult women with self-reported BMI >25 kg/m^2^	58	0	42	2 months	Body weight
Laing, 2014 [20]	United States	RCT	Primary care patients with BMI >25 kg/m^2^	212	27	43.3	6 months	Body weight
Glynn, 2014 [21]	Ireland (West)	RCT	Primary care patients	90	36	44	2 months	Body weight, BMI, step count
Smith, 2014 [22]	Australia	RCT	Adolescent boys in low-income communities	361	100	12.7	20 weeks	BMI, waist circumference
Hebden, 2014 [23]	Australia	RCT	Students and staff, Australian University	41	19	22.6	3 months	Body weight, BMI, MPA^g^, VPA^h^
Partridge, 2015 [24]	Australia	RCT	Participants at risk of excess weight gain	250	38	27.2	9 months	Body weight, BMI, MPA, VPA

^a^SS: sample size.

^b^CCS: case-control study.

^c^N/A: not applicable.

^d^BMI: body mass index.

^e^RCT: randomized controlled trial.

^f^MCCT: matched case-control trial.

^g^MPA: moderate physical activity.

^h^VPA: vigorous physical activity.

**Table 2:** Characteristics of intervention types and description of apps.

Author, year	Outcome	Intervention type	Description of app	Control group treatment
Lee, 2010 [13]	Body weight, BMI^a^	Mobile phone app + usual care	Smart Diet app: provides a personalized diet profile; promotes knowledge about nutrition using a diet game	Not described
Turner-McGrievy, 2011 [14], 2013 [15]	BMI, physical activity	Mobile phone app + podcast + Twitter messages	Diet and physical activity-monitoring app (2010 version of FatSecret’s Calorie Counter app)	Podcast only
Kirwan, 2012 [16]	Physical activity	Mobile phone app + 10,000 steps program	Self-monitoring and self-reported physical activity levels (iStepLog)	10,000 steps program
Carter, 2013 [17]	Body weight, BMI, physical activity	Mobile phone app	Self-monitoring with managed intervention delivered by (MMM) app	Self-monitoring by using a food diary + a calorie-counting book
Allen, 2013 [18]	Body weight, BMI, waist circumference	Mobile phone app + intensive counseling	Lose It! (weight-loss app)	Intensive counseling
Brindal, 2013 [19]	Body weight	Mobile phone app + Celebrity Slim program	Support app: My Meals + My Weight + My Task	Celebrity Slim program alone
Laing, 2014 [20]	Body weight	Mobile phone app + usual care	MyFitnessPal app	Counseling about activities to lose weight + one-page educational handout on healthy eating
Glynn, 2014 [21]	Body weight, BMI, step count	Mobile phone app + usual care	Accupedo-Pro Pedometer app	Physical activity goals and information on the benefits of exercise + Be active physical activity promotion brochure
Smith, 2014 [22]	BMI, waist circumference	Mobile phone app + parent newsletters, seminars, sport sessions, lunchtime physical activity-mentoring sessions, teachers attend two 6-h workshops, and one fitness instructor session	Physical activity monitoring, recording of fitness challenge results, tailored motivational messaging, goal setting for physical activity and screen time	Traditional intervention (ie, regularly scheduled school sports and physical education lessons)
Hebden, 2014 [23]	Body weight, BMI, MPA^b^, VPA^c^	Mobile phone app + SMS^d^text messages + email messages + Internet forums + usual care	Four mobile phone apps per behavior	A 10-page printed booklet
Partridge, 2015 [24]	Body weight, BMI, MPA, VPA	Mobile phone app + SMS text messages + email messages + Internet forums + community blog + usual care	Mobile phone apps that provide education and allow self-monitoring	Control participants received a mailed two-page handout, four text messages, and access to a website

^a^BMI: body mass index.

^b^MPA: moderate physical activity.

^c^VPA: vigorous physical activity.

^d^SMS: short message service.

### Meta-Analysis of Mobile App Intervention and Body Weight

**Figure 2:**
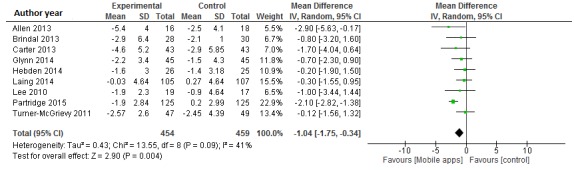
Meta-analysis of the net change in body weight (kg) associated with mobile phone app intervention, expressed as the change during the mobile phone app intervention minus the change during the control diet. The area of each square is proportional to the inverse of the variance of the weighted mean difference. Horizontal lines represent 95% CIs. Diamonds represent pooled estimates from inverse variance (IV) weighted random-effects models.

Data from 913 participants were analyzed in nine clinical trials [13,14,17-21,23,24]. Compared with the control group, mobile phone app interventions resulted in significant decreases in body weight, with the pooled estimates of the net change in body weight being -1.04 kg (95% CI -1.75 to -0.34; I2 = 41%) (see Figure 2). In the sensitivity analysis, we excluded the Lee study [13] because it was not a randomized study and did not include any intervention in the control group. The exclusion of this study did not modify the results (WMD -1.04 kg, 95% CI -1.80 to -0.27 kg; I2 = 48%).

The funnel plot showed reasonable symmetry, which suggested no evidence of publication bias in the clinical trials of mobile apps for weight loss (see Multimedia Appendix 2). In the sensitivity analysis, the exclusion of individual studies did not substantially modify estimates; the pooled WMDs ranged from -0.63 to -1.20 kg.

### Meta-Analysis of Mobile App Intervention and Body Mass Index

**Figure 3:**
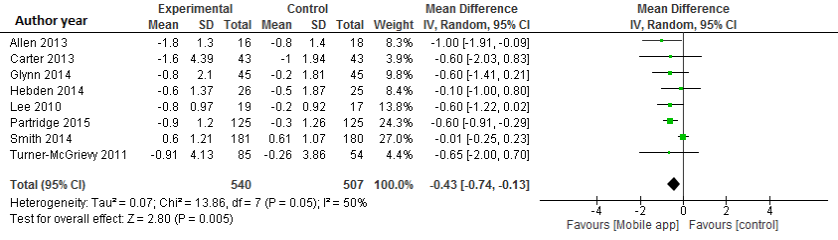
Meta-analysis of the net change in BMI (kg/m^2^) associated with mobile phone app intervention, expressed as the change during the mobile app intervention minus the change during the control diet. The area of each square is proportional to the inverse of the variance of the weighted mean difference. Horizontal lines represent 95% CIs. Diamonds represent pooled estimates from inverse variance (IV) weighted random-effects models.

Data from 1047 participants were analyzed in eight clinical trials [14,17,18,21-24]. Pooled results indicated a significant net difference in BMI between mobile phone app and control intervention groups (WMD -0.43 kg/m^2^, 95% CI -0.74 to -0.13; I2 = 50%) (see Figure 3). The exclusion of the Lee study [13] did not modify the results (WMD -0.42 kg/m^2^, 95% CI -0.76 to -0.07; I2 = 54%).

The funnel plot showed reasonable symmetry, which suggested no evidence of publication bias in the clinical trials of mobile apps for weight loss (see Multimedia Appendix 2). In the sensitivity analysis, the exclusion of individual studies did not substantially modify estimates; the pooled WMDs ranged from -0.36 to -0.59 kg/m^2^.

### Meta-Analysis of Mobile App Intervention and Physical Activity

**Figure 4:**
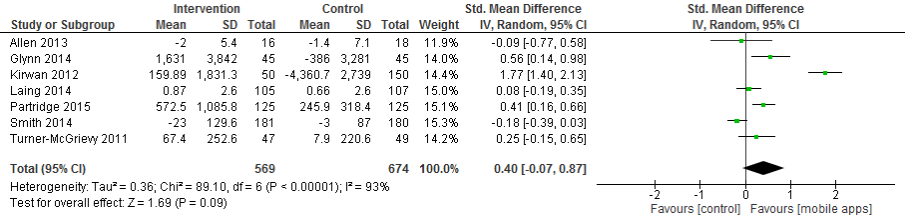
Meta-analysis of the net change in physical activity associated with mobile phone app intervention, expressed as the change during the mobile app intervention minus the change during the control intervention. The area of each square is proportional to the inverse of the variance of the standardized mean difference. Horizontal lines represent 95% CIs. Diamonds represent pooled estimates from inverse variance (IV) weighted random-effects models.

Data from 1243 participants were analyzed in seven clinical trials [14,16,18,20-22,24]. Pooled results indicated a nonsignificant difference in physical activity between mobile app and control intervention groups (SMD 0.40, 95% CI -0.07 to 0.87; I2 = 93%) (see Figure 4). The sensitivity analysis indicated that no single study was the main origin of heterogeneity between studies. Next, we excluded any two studies in turn and pooled the data of the remaining studies. The heterogeneity was decreased (I2 = 33%) after two studies—Kirwan et al [16] and Smith et al [22]—were excluded (SMD 0.27, 95% CI 0.08-0.47).

The funnel plot showed reasonable symmetry, which suggested no evidence of publication bias in the clinical trials of mobile apps designed to increase physical activity (see Multimedia Appendix 2). In the sensitivity analysis, the exclusion of individual studies did not modify the estimates; the pooled SMDs ranged from 0.17 to 0.51.

### Risk of Bias in Included Studies

**Figure 5:**
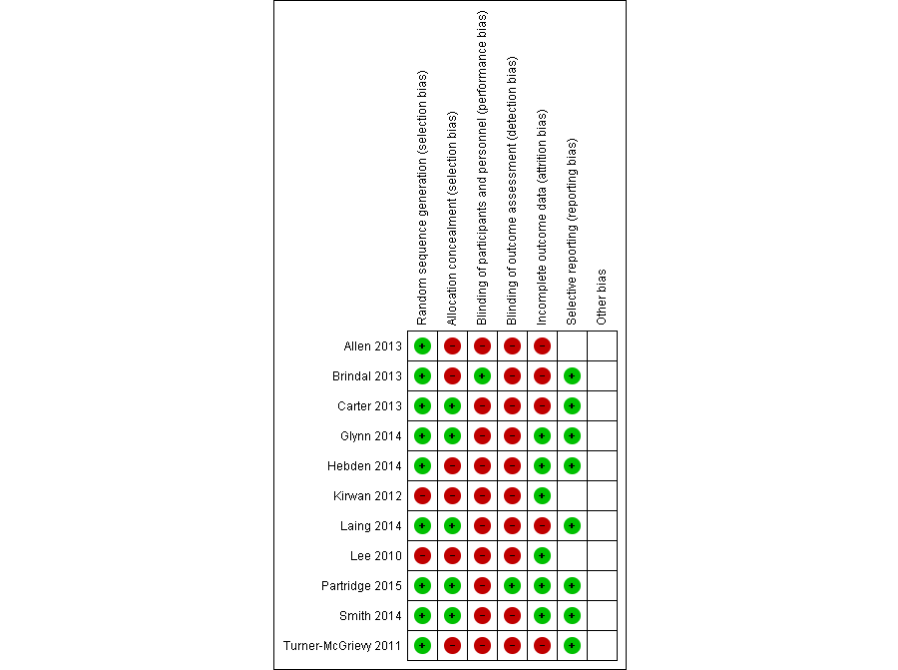
Summary of review authors’ assessments of risk of bias for each Cochrane item and each included study.

Randomization was considered adequate in most of the studies (see Figure 5). Only one study's participants were blinded as to their allocations [19], and in another study [24] the research staff collecting data on outcomes were blinded to the allocation of participants. For most of the studies we located the original study protocols [15,17,19-24]. Since we found no discrepancies between the outcomes that authors originally intended to measure and those reported in this study, we judged the risk of reporting bias to be low for this domain.

## Discussion

The current meta-analysis suggested that mobile phone app interventions compared with various control interventions significantly reduced body weight by 1.04 kg, reduced BMI by 0.43 kg/m^2^, and nonsignificantly increased physical activity by an SMD of 0.40. Our findings were robust across sensitivity analyses. Although the mean reductions in body weight and BMI were modest, it would not be expected for a single change in weight-loss interventions, such as mobile phone apps, to cause clinically meaningful weight loss compared with other control interventions [25]. Many of the control group treatments were other interventions.  This could dilute the analysis, as it is possible that in some of the studies the treatment group showed a significant change, while the control group also showed a similar significant result.  In our sensitivity analyses, the results were not modified when we excluded one study that did not describe if the control group had received any intervention [13].

Some of the most recognizable research in mobile interventions has focused on text-messaging interventions, or SMS. A previous meta-analysis [26] found that mobile phone interventions were associated with significant changes in body weight and BMI compared with the control group (-1.44 kg and -0.24 units, respectively). This meta-analysis included only mobile interventions based on contacts by SMS and multimedia message services (MMS). A previous systematic review found strong evidence from the included RCT that weight loss occurs in the short term because of mobile technology interventions [27]. Another systematic review that included seven articles demonstrated a beneficial impact of text messaging or a mobile app for reducing physical inactivity and/or overweight/obesity [28]. Finally, a recent systematic review found that most apps that are focused on weight loss have inconsistent outcomes [29].

To our knowledge, this study is the first meta-analysis to summarize the evidence to date regarding effects of mobile phone app interventions compared with various control interventions. We excluded from our meta-analysis interventions based only on text messaging and focused solely on mobile phone apps because text-message interventions do not utilize the full potential of mobile phone technologies. Well-designed apps expand the potential for technology-based health interventions to impact populations in ways that previously were not possible and cannot be achieved without the capabilities of mobile phone software. Therefore, the need to regulate this growing market is becoming a concern, with increased advertising claims about effectiveness and researchers emphasizing the need for studies that will contribute scientific evidence about the true impact of these types of apps. The portability of mobile phones enables users to have access 24 hours a day, making possible the long-term management and reinforcement of health behaviors through a variety of communications and apps. Fitness and weight-loss mobile phone apps allow for the tracking of diet, weight, and physical activity; making grocery and restaurant decisions; cooking healthy meals [28]; or gamification of the intervention. Moreover, participants do not need to carry an extra piece of purchased technology, such as a pedometer, to track physical activity.

Several limitations have been noted. Only a small number of available studies assess the effectiveness of mobile phone apps in weight-loss programs, and they included small sample sizes and short follow-up periods. The use of apps for improving physical activity and reducing anthropometric measures is relatively new. More randomized controlled trials with larger sample sizes and longer follow-up periods are needed to determine the effectiveness of mobile apps in improving health outcomes.

A recent study aimed to evaluate diet/nutrition and anthropometric apps based on incorporation of features consistent with theories of behavior change; all apps were found to be very low in theoretical content or use of theory to guide behavior change [28]. The studies included in this meta-analysis also varied in the content and theoretical basis of the intervention. Further investigation into the effective features of the mobile phone apps and the interventions’ consistencies with theories of behavior change was not possible; this should be considered an area for future research.

The risk of bias was high in most of the studies and future research should improve on several issues, such as the use of blinding or improving attrition rate. All studies, except for one [19], failed to conceal the blinding of participants and personnel, and only one study [24] blinded the research staff collecting the data on outcomes as to the allocation of participants. Given the nature of the intervention, blinding of participants and personnel is difficult. However, it is important to recognize the possible influence of patient and personnel expectations. Therefore, adoption of blinding techniques, such as the use of sham procedures, blinding participants to the study hypothesis, or using a blinded centralized assessment of primary outcomes, will improve the quality of the evidence [30].

A large attrition rate was noted in some of the studies [17,20] included in this meta-analysis. High attrition rates are common in weight-loss interventions, and the reasons for this are likely complex and varied [31]. Attrition has an obvious impact on the validity of results obtained and can introduce bias; for example, those more motivated to reduce their weight or increase physical activity may remain in the trial. Moreover, several studies found that most participants rarely used the app after the first month of the study [20,23]. As with other weight-loss interventions, the most effective app may be one that can engage people for the longest period. It is known that adherence to self-monitoring of food intake is associated with twice as much weight loss as infrequent monitoring [32]. Without the participant’s active engagement, the app is not likely to be used and as a result will not be effective [28]. The most highly ranked engagement strategies identified are (in order of preference) ease of use, design aesthetic, feedback, function, ability to change design to suit own preference, tailored information, and unique mobile phone features [33]. Weight-loss apps may need to be substantially more engaging or less time-consuming to produce weight reduction in the average individual. It would be useful to design strategies to increase “app appeal” before implementing this type of intervention. Gamification of the app, financial incentives, or delivering the app in a setting of group competition could be important adjuncts to increase motivation to use the app and lose weight [20,34].

To our knowledge, this is the first meta-analysis to summarize the effectiveness of mobile apps designed to improve physical activity and reduce anthropometric measures. Our meta-analysis highlighted the need to perform larger, high-quality, randomized controlled clinical trials with longer follow-up. The number of available mobile phone apps is growing steadily, and mobile phones are constantly undergoing updates so the features have changed over time. Incorporating features consistent with theories of behavior change into health-related apps would be useful to improve weight-loss outcomes [35]. We searched several databases in order to avoid publication bias, which is a concern in meta-analyses that only include published studies. Using funnel plots in our meta-analysis made it possible to exclude publication bias with some confidence.

As the world’s understanding of health and how to empower individuals to take better care of their health changes, health professionals must treat this change as progress. However, we must ensure that the patient is using a mobile app with appropriate quality guarantees. This meta-analysis aimed to provide a rigorous, systematic, and quantitative review of the studies that have analyzed the effectiveness of mobile apps and attempted to measure their influence on lifestyle changes. Bombarded by information overload in all arenas, health professionals and managers have a need for the insights provided by a tool like the meta-analysis; this could help them make decisions and decide what direction we should be moving in our efforts to promote weight loss, increase physical activity, and confront the public health crisis presented by overweight and obesity.

In summary, the results from this meta-analysis demonstrated that interventions based on mobile phone apps are associated with more weight loss than other types of interventions. Furthermore, a nonsignificant increase in physical activity was detected. Evidence form this meta-analysis shows that mobile phone app-based intervention may be useful tools for weight loss.

## Appendix

Multimedia Appendix 1 Database search strategies.

Multimedia Appendix 2 Funnel plots from the meta-analysis of the association of mobile phone app intervention with (A) body weight change (kg), (B) body mass index (kg/m2), and (C) physical activity. SE, standard error.

## Abbreviations

BMI: body mass index
CCS: case-control study
CINAHL: Cumulative Index to Nursing and Allied Health Literature
IDIAP: Institut Universitari d'Investigació en Atenció Primària
ITU: International Telecommunications Union
MCCT: matched case-control trial
MMS: multimedia message service
MPA: moderate physical activity
N/A: not applicable
PDA: personal digital assistant
RCT: randomized controlled trial
SDpost: SD at the end of the intervention
SDpre: SD at baseline
SMD: standardized mean difference
SMS: short message service
SS: sample size
VPA: vigorous physical activity
WMD: weighted mean difference
