# Supplementary material for: Mobile Phone Apps to Promote Weight Loss and Increase Physical Activity: A Systematic Review and Meta-Analysis
Source: J Med Internet Res. 2015 Nov 10;17(11):e253. doi: 10.2196/jmir.4836 (PMC4704965; doi:10.2196/jmir.4836)
Supplement: Multimedia Appendix 1 [file jmir_v17i11e253_app1.pdf]

Appendix I: Database search strategies

**Database**	**Search strategy**
PubMed	("mobile application" OR apps OR smartphone) AND ("physical activity" OR exercise OR activity OR inactivity OR weight OR obesity OR "body mass index" OR "waist circumference" OR "body weight") AND "humans"[MeSH Terms]
CINAHL	(MH "Mobile Applications") OR ((Mobile AND Application*) OR app OR apps OR smartphone*) AND (MH "Weight Reduction Programs") OR (MH "Waist Circumference") OR weight OR (waist AND circumference*) OR (MH "Body Mass Index") OR "body mass" OR ((motor OR physical OR locomotor) AND activit*) OR exercise OR inactivit* OR obesity OR (MH "Obesity+") OR (MH "Body Weight+") OR (MH "Motor Activity+") OR (MH "Body Mass Index")
Scopus	(TITLE ((mobile AND application*) OR app OR apps OR smartphone*) AND TITLE ((waist AND circumference*) OR "Body Mass" OR ((motor OR physical OR locomotor) AND activit*) OR exercise OR inactivit* OR "Obesity" OR "Body Weight" OR weight*))
