# Supplementary figures and images for: Mobile Phone Apps to Promote Weight Loss and Increase Physical Activity: A Systematic Review and Meta-Analysis
Source: J Med Internet Res. 2015 Nov 10;17(11):e253. doi: 10.2196/jmir.4836 (PMC4704965; doi:10.2196/jmir.4836)

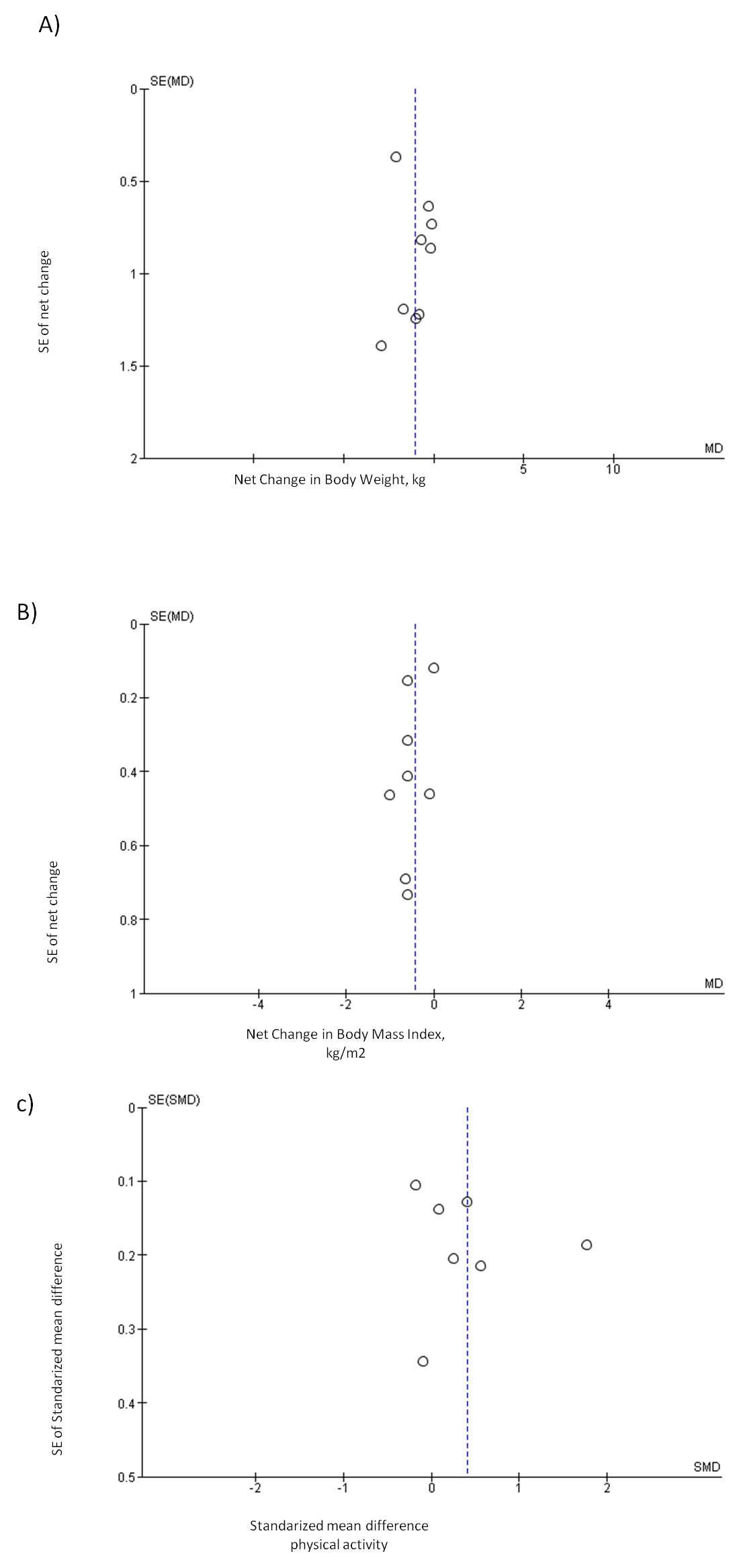

Supplement: Multimedia Appendix 2 [file jmir_v17i11e253_app2.png]
